# From rhetoric to reality: organisational practices of health equity in Switzerland

**DOI:** 10.1186/s12939-025-02688-9

**Published:** 2025-11-28

**Authors:** Jodie Freeman, Rosa Emilia Henn, Annika Frahsa

**Affiliations:** Institute of Social and Preventive Medicine, University of Bern, Bern, Switzerland

## Abstract

Despite growing international consensus on the importance of addressing health inequities, the concept of health equity continues to evolve in its definition, relevance, and application across global contexts. While its prominence in international discourse and strategic frameworks has grown, the ways in which health equity is interpreted, prioritised, and operationalised within health-related organisations remain uneven and insufficiently examined. We conducted a qualitative exploratory study, using in-depth semi-structured interviews with representatives (*n* = 16) from public health authorities, non-governmental organisations, and academic institutions in Switzerland to explore divergent understandings, current initiatives, perceived challenges, and success measures related to health equity. We conducted Thematic Analysis (TA), supported by NVivo software. We identified four overarching themes: conceptual understandings of health equity, framing health equity in organisational discourse and strategy, challenges in operationalising health equity, and strategies to better implement health equity. The main findings reinforce that advancing health equity is not merely a technical challenge but a deeply political and relational one. Shared language should be accompanied by structural alignment; measurement should be enabled by meaningful data; and vision should be grounded in long-term, collaborative practice. Institutions that succeed in health equity do so not because of isolated actions, but because of a consistent, system-wide commitment. Operationalising health equity requires continuous professional reflexivity and strong local networks to address structural barriers and evolving disparities with flexible, ongoing action. In the absence of political continuity, robust metrics, and actionable data, health equity risks tend to be deprioritised. Embedding equity into routine practice and making its impact visible is critical to sustained progress.

**Supplementary Information:**

The online version contains supplementary material available at 10.1186/s12939-025-02688-9.

## Introduction

Despite growing international consensus on the importance of addressing health inequities, the concept of health equity continues to evolve in its definition, relevance, and application across global contexts [1]. Health equity-broadly understood as the absence of avoidable, unfair, and remediable differences in health among population groups- has become a central concern in global public health [2, 3]. While its prominence in international discourse and strategic frameworks has grown, how health equity is interpreted, prioritised, and operationalised within health-related organisations remain uneven and insufficiently examined [4]. The core principle of health equity is to ensure fair access to health resources regardless of socioeconomic status, geography, or ethnocultural background, is widely acknowledged [5]. Yet, in many cases, it remains a theoretical ideal rather than a practical reality, unless it is translated into deliberate organisational strategies and supported by coherent, system-wide policies.

Health equity is a key concept in policy and strategic discourse. Its realisation, though, depends on how organisations conceptualise and operationalise it [1, 4, 6]. This includes the language they adopt, the issues they prioritise, the data they collect and use, and the strategies they implement. Often conflated with equality, equity instead focuses on addressing the structural and social determinants of health that systematically disadvantage certain groups [7]. Some research even openly acknowledges using the terms ‘equity’ and ‘equality’ interchangeably, despite their distinct meanings and implications [1]. Equality tends to be perceived as a less value-laden concept that refers to providing the same resources or opportunities to all individuals, whereas equity is an explicit normative concept that emphasises fairness and justice by allocating resources based on specific needs and to design processes so that they achieve comparable outcomes [8].

Relatively few studies have examined how organisational actors themselves understand and embed equity within internal processes, strategic planning, and institutional culture. Among those that do, most are rooted in specific national contexts, often within high-income countries committed to universal health coverage. A Canadian study of primary health care organisations explored how equity principles were translated into practice in centres serving highly marginalised, predominantly Indigenous communities [9]. Although this research offers important insights, the context defined by deep structural disadvantage, historical trauma, and targeted service focus limits its transferability to more heterogenous or decentralised health systems.

Taken together, these studies highlight the importance of conceptual clarity, resource allocation, and cross-sectoral partnerships for achieving equity. Yet they also point to a persistent gap: how equity is interpreted, operationalised, and institutionalised within everyday organisational settings, especially in systems that lack a national framework for health equity or operate across culturally diverse regions. These institutional dynamics represent an emerging and highly relevant area for research, as they shape whether equity principles translate into sustainable change within health systems [10, 11].

Organisational actors play a pivotal role in translating health equity goals into actionable strategies [11]. Their conceptualisations of equity, the language they use, the data they collect, and the partnerships they form all have concrete implications for the communities they serve. Understanding how these organisations make sense of and act on equity is therefore critical to identifying barriers to progress and enabling more effective, context-sensitive interventions. However, there remains a limited focus on the organisational relevance and practical application of health equity [12].

Switzerland offers a unique context to explore health equity in-depth. As a high-income nation with a decentralised, federalist health system and high overall health standards, it nonetheless experiences health inequities shaped by socioeconomic and migration-related factors [13]. This makes Switzerland a compelling site for further investigation how health equity is taken up at the institutional level, particularly due to its linguistic and sociocultural diversity and its strong emphasis on local governance. The Swiss national health strategy declares that the health system is to be based on openness and solidarity, accessible for everyone, independent from language, origin, social and educational status. However, amid this commitment to equity, limited insights exist on how public health authorities, academic institutions, and Non-Governmental Organisations (NGO) sectors within Switzerland understand and implement equity in practice. While existing research in Switzerland has addressed structural and policy aspects of the health system, most existing studies tend to focus on the health system structure and policy, health outcomes, disparities and access to care [14–16]. However, studies that directly examine how health organisations in Switzerland conceptualise, prioritise and operationalise health equity remain scarce.

This study seeks to address this gap by exploring how key health-related institutions in Switzerland conceptualise, prioritise, and implement health equity within their work. It critically examines organisational understandings of health equity in the Swiss context. It adopts a community-level lens to explore how equity is framed, measured, and acted upon. By doing so, it aims to shed light on the barriers and enablers of progress towards more equitable health systems. Drawing on qualitative interviews with representatives from public health authorities, academic institutions, foundations and NGOs, we examine how equity is understood across organisational contexts, how it is framed in discourse and strategy, and what practical challenges and enablers influence its operationalisation. In doing so, we aim to contribute to a deeper understanding of the organisational dimensions of health equity, offering insights relevant to policy, practice, and future research across similarly complex health systems.

We sought to explore four research questions: (1) how health equity is conceptualised by health-related organisations in Switzerland, (2) how health equity is framed in organisational practice and discourse, (3) what practices, strategies and assessments support or hinder the implementation of health equity, and (4) what structural, cultural or systemic barriers affect the operationalisation of health equity in the Swiss context.

## Methods

We conducted a qualitative exploratory study, using Thematic Analysis of in-depth semi-structured interviews with representatives from public health agencies/authorities, NGOs, and academic institutions in Switzerland.

### Participants

**Fig. 1:**
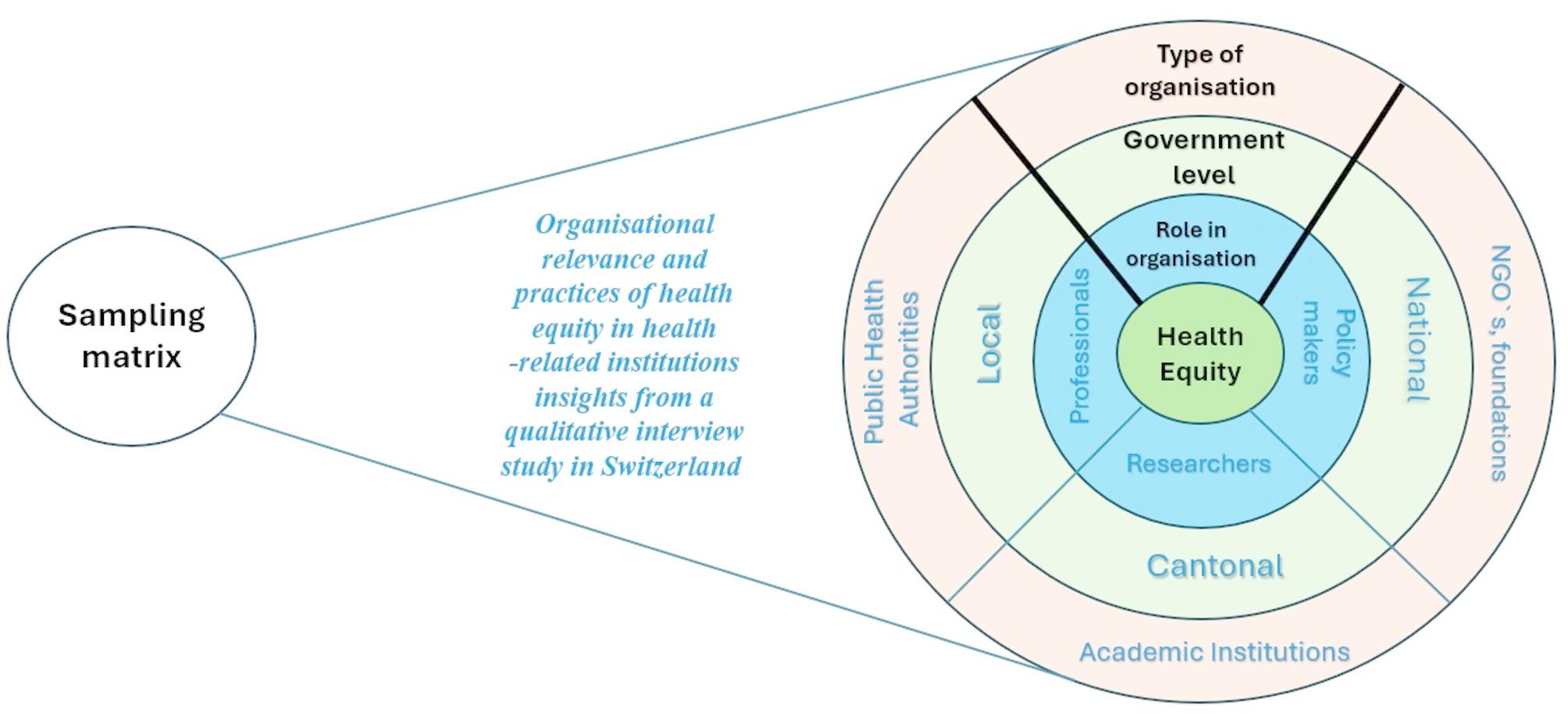
Sampling matrix

We used purposive sampling to recruit participants who varied across key dimensions of the organisational context in Switzerland: organisational type, government level, and participants’ professional role (cf. Fig. 1). Inclusion criteria required participants to hold a leadership, coordination, or specialist role with direct experience in health equity efforts. Eligible organisations included public health authorities, NGOs, foundations, or academic institutions operating at local, cantonal, or national levels.

We also selected participants based on their capacity to reflect on both organisational strategies and broader systemic challenges. Participants were identified through existing networks and/or public records and were invited via email. Snowball sampling was also used to recruit additional participants through expert recommendations during interviews.

### Data generation

The first author conducted and audio-recorded the interviews from March-May 2025. A comprehensive interview guide (see Appendix 1) was used to facilitate discussion. The interview questions were designed to stimulate discussions on how health equity is understood and conceptualised. Interviews were conducted in either German or English depending on participant’s preferences. The participants’ roles in the organisation and length of employment within the health system and their current area of work were also captured during the interviews. Interviews ranged in duration from 39 min to 81 min, with a mean length of approximately 60 min. Data collection was guided by the concept of *information power* [17], which suggests that the adequacy of a sample is determined not by size alone but by the richness and relevance of the data in addressing the research question.

### Data analysis

All interviews were audio-recorded and transcribed verbatim. Transcripts originally in German were translated into English, with close attention to preserving meaning and contextual nuances. The first author JF led the analysis and immersed themselves in the data by reading each transcript multiple times, making detailed notes to capture initial impressions and emerging insights [18, 19]. In the second phase, JF generated initial codes to identify meaningful features within the data, using NVivo (NVivo, QSR International, 2015) and applied both descriptive semantic and more latent interpretative approaches. In the third phase, JF systematically organised codes into potential themes, enabling the identification of broader patterns of meaning across the dataset. Given the multidisciplinary expertise of the author team, reflexivity was embedded throughout the study; a detailed account of reflexive practices and their influence on the research process is provided in Appendix 2. In phase four, we reviewed and refined themes to ensure they were coherent, distinct, and meaningful in relation to the entire data set. In phase five, the authors collaboratively defined and named each theme, articulating the core story and message it conveyed, as well as its relevance to the overall narrative. Finally, in phase six, we wove themes into an analytical narrative, illustrated with vivid and representative quotes from participants [18, 19]. This narrative demonstrated how the themes addressed the research questions and situated the findings within the broader academic literature.

## Findings

As shown in Table 1, a total of 16 individuals participated in this study (*n* = 14 individual interviews, and *n* = 2 in a joint interview). Participants included 5 academics, 2 representatives from NGOs, 1 representative from a Swiss foundation and 8 participants from public health authorities. 3 organisations declined to take part due to time restrictions. 11 organisations did not respond to the invitation or email reminder.

**Table 1:** Recruitment matrix

	Type of organisation	Role in organisations	Level of Government
P01	Public health authority ^*(PH1)*^	Leadership	Cantonal
P02	Public health authority ^*(PH2)*^	Coordination	Cantonal
P03	Public health authority ^*(PH3)*^	Leadership and coordination	Cantonal
P04	NGO ^*(NGO1)*^	Leadership	Local
P05	Academic institution ^*(AI1)*^	Leadership	Cantonal
P06	Academic institution ^*(AI2)*^	Leadership	National
P07	Academic institution ^*(AI3)*^	Leadership	National
P08	Academic institution ^*(AI4)*^	Leadership	National
P09	Public health authority ^*(PH4)*^	Specialist	Cantonal
P10	Foundation ^*(F1)*^	Leadership	National
P11	NGO ^*(NGO2)*^	Coordination	Cantonal
P12	Public health authority ^*(PH5)*^	Leadership and coordination	Cantonal
P13	Public health authority ^*(PH6)*^	Coordination	Cantonal
P14	Academic institution ^*(AI5)*^	Leadership	National

**Fig. 2:**
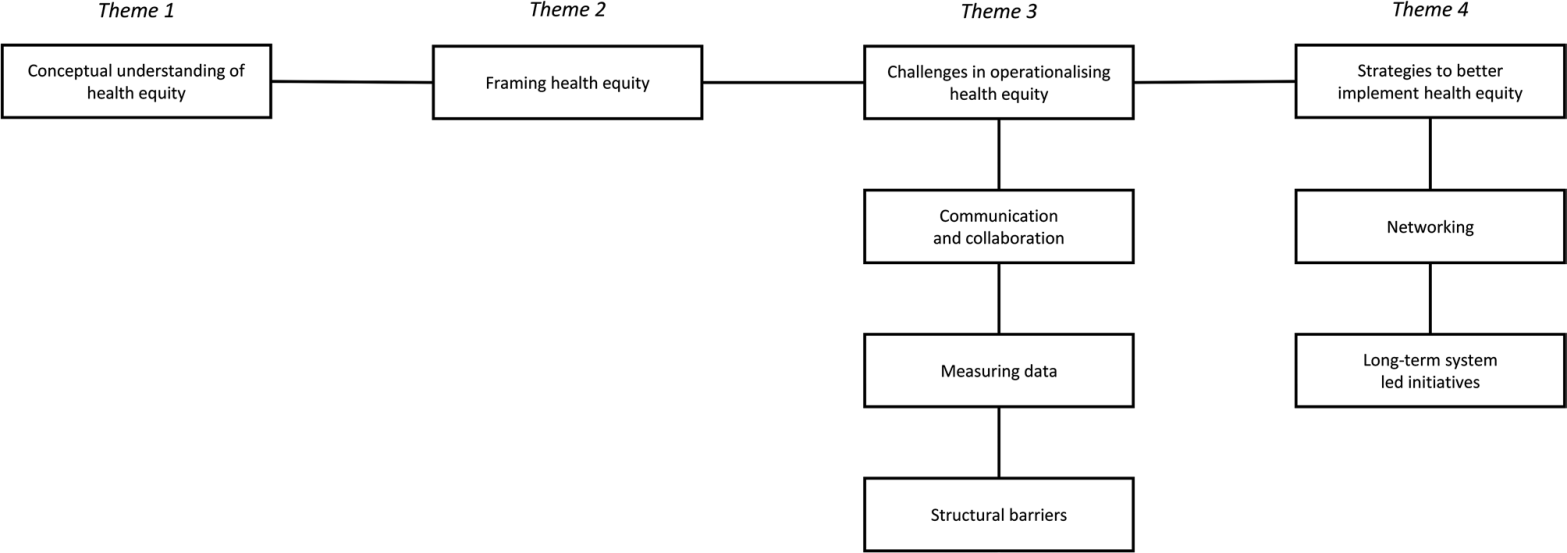
Thematic map

Based on participants’ accounts, we identified four overarching themes that illuminate how health equity is conceptualised, valued, and operationalised across different institutional contexts: *(1) Conceptual understandings of health equity*,* (2) Framing health equity in organisational discourse and strategy*,* (3) Challenges in operationalising health equity*,* (4) Strategies to better implement health equity* (cf. Figure 2).

The theme *Challenges in operationalising health equity* was further divided into three distinct subthemes: communication, measuring data, and structural barriers. Similarly, the theme *Strategies to better implement health equity* was split into two subthemes: networking and long-term system-led initiatives. In the following sections, we explore each theme in detail, using verbatim excerpts to capture the depth and complexity of stakeholder perspectives.

### Conceptual understandings of health equity

The conceptual understanding of health equity refers to how the term is defined and interpreted, an understanding that varies across organisational types. This theme primarily explores how health equity is linked to or differentiated from related concepts such as equality, fairness, and justice. Academic institutions tended to discuss equity as a matter of fairness, emphasising the need for proactive measures to address structural disadvantages. Their interpretations clearly distinguished equity not only from equality but also from the notion of equal opportunity. As one leading academic explained:

*There are big differences between equity*,* equality*,* and opportunity*^*(AI5)*^

*Having a diverse workforce doesn’t automatically mean it’s an equitable one* ^*(AI4)*^

Concerns were raised that ‘diversity’, in this case, the presence of individuals from varied backgrounds, is sometimes employed superficially as a symbol of progress or fairness, without addressing the deeper, more fundamental issues of equity. Specifically, questions remain about whether these individuals are genuinely supported, respected, and empowered within their organisations.

Public health institutions also demonstrated a clear conceptual distinction between equity and equality, often describing health equity as a foundational principle embedded across all their programs and initiatives.

*I mean it’s about providing the necessary resources that different people need*,* not everyone needs the same things or the same amount so it’s about identifying what people*,* a particular group needs and providing that chance for them*^*(PH6)*^

In contrast, although representatives from two NGOs recognised the theoretical distinction between equality and equity, this understanding was not consistently reflected in their practices or operational decisions. For example, participants described tailoring interventions to community needs in principle, yet in practice reported applying the same distribution of resources or programs to all communities regardless of differing needs, highlighting a gap between discourse and action.

### Framing health equity in organisational discourse and strategy

Beyond conceptual understandings, *framing* of health equity was very prominent, referring to how health equity is presented, embedded, and communicated in internal discourses, external messaging, and policy engagement. The framing of health equity varied significantly not only between the three organisational types but also within them, influenced by institutional cultures, strategic priorities and the professional role held in an organisation.

In academic settings, health equity was often framed as a *societal and political responsibility*. One leading academic emphasised the ongoing nature of equity challenges and the need for continuous effort and policy engagement.

*Health equity is an ongoing societal challenge. Even when progress is made*,* new inequities emerge*,* requiring continuous effort and policy.*^*(AI1)*^

Another lead academic framed health equity as a moral imperative, whilst also discussing it as a legal issue, highlighting it might be even more decisive to show health equity as a cost-effective necessity.

*One of my jury members was a lawyer and argued that it’s not only moral but legal… Personally*,* I think [the legal aspect] is less important… but we need to show that it’s also cost-effective. Treating everyone fairly*,* I mean.*^*(AI3)*^

This participant explained that health equity was frequently mentioned in discussions but often only given token acknowledgement. Although it appeared in conversations, it rarely led to meaningful actions or real change.

*the majority just give it lip service*,* they talk about it enough*,* so it looks important but then nothing ever changes*^*(IA3)*^

Among NGOs, framing health equity was closely tied to organisational ethos and strategic goals. It was often positioned as part of a broader mission related to inclusion, access, or social justice, though how explicitly health equity featured depended on the culture and capacity of the organisation. Similarly, a public health professional stressed that the framing of health equity tended to be often shaped by the needs of individuals, the public and the community as a whole.

*How we talk about equity really depends on who we’re speaking to*,* what we want to achieve*,* and the context we’re in.*^*(NGO1)*^

Importantly, several participants across academic and public health sectors highlighted the narrow definitions of equity in practice. This revealed which dimensions of equity tended to be prioritised or overlooked within institutions and policies.

*Here*,* equity is currently mostly understood as gender equity and not much more. Maybe disability is included in teaching. But migration*,* for example*,* is not*,* it’s just not on the radar.*^*(PH6)*^

These findings highlighted that the framing of health equity is not merely conceptual but plays a crucial role in shaping organisational priorities, influencing stakeholder engagement, and determining which populations and structural drivers are seen as relevant to equity work.

### Challenges in operationalising health equity

This theme explores the challenges, both within and across organisational settings. It is split up into 3 distinct sub themes: communication and collaboration, measuring data and structural barriers.

#### Communication and collaboration

Notably, several academic stakeholders expressed concern about a perceived reluctance among organisations to collaborate, despite the recognised benefits of transparency and information sharing.

One academic attributed this hesitation to cultural factors within the Swiss context, describing a conservative and compartmentalised working culture that may hinder inter-organisational cooperation. One interviewee used the German term “Gärtchendenken”, a metaphorical expression that literally means “little garden thinking” - everyone tending their own garden without ever looking over the fence to see how their work affects others or could benefit from cooperation. The term tends to be used critically, especially in organisational, political, or academic contexts, when referring to narrow-minded, territorial, or siloed way of thinking, where individuals or groups focus only on their own small area of responsibility or interest and fail to collaborate or consider the bigger picture [20].

*This is the kind of thinking in Switzerland—we call that ‘Gärtchendenken* ^*(AI1)*^

Another lead academic noted that while they occasionally collaborated with other institutions, they often were not aligned, especially when it came to understanding the core principles of health equity and its practical significance.

*Often*,* we are not on the same page when it comes to what achieving health equity means in practice*,* even if we all have the same definitions*^*(AI4)*^

One representative of a public health institution described extensive collaboration with the local community and service providers; however, interaction with other larger public health institutions was limited. This highlighted a gap in information sharing within the network, particularly for knowledge that extends beyond what is already publicly available.

#### Measuring data

A reoccurring challenge identified as a barrier to promoting and advancing health equity was the lack of robust data and the associated challenges of measurability. While some academic institutions and public health organisations conducted evaluations related to health equity, all participants agreed that this area remained significantly underdeveloped. Even in cases where formal assessments existed, primarily within academic settings, the absence of comprehensive data impeded the ability to demonstrate the impact of health equity-related practices, programs, and policies.

Moreover, the lack of predefined assessment criteria further complicated measurement efforts. Several participants emphasised that without clear indicators, it became difficult to quantify progress. One professional noted:

*It only becomes measurable when we have numbers. And in order to have numbers*,* we also have to define criteria.*^*(F1)*^

The issue was compounded by national-level data limitations. Participants highlighted the absence of routine collection of key demographic indicators, such as ethnic or migration background:

*In Switzerland*,* we have almost no data on anything. We don’t collect data on ethnic background or migration background.*^(PH3)^

As a result, understanding the effects of interventions was hindered by limited evidence:

*That’s a problem. Which means we have very little data*,* and that makes it hard to say what effect interventions actually have.*^*(AI1)*^

#### Structural barriers

A consistently emphasised theme across stakeholder interviews was the critical need for strong, sustained inter- and intra-organisational connections, grounded in transparency and shared commitment, to mitigate the disruptive effects of *policy volatility* and *leadership turnover*. Participants widely acknowledged that frequent changes in government leadership, policy direction, or institutional priorities often reshaped how health equity was understood, framed, and operationalised within organisations.

As one academic participant observed:

*Whenever the government changes*,* the policies and conditions change too.*^*(AI4)*^

*The national policy program on health equity in Switzerland has ended*,* leading to less public and political attention on the topic*^*(AI3)*^

These changes could lead to a loss of institutional knowledge, particularly when equity-driven work was not structurally embedded but instead tied to specific individuals or programs. A public health professional highlighted this fragility:

*When they leave*,* sometimes the knowledge goes with them*,* and priorities change again.*^*(PH3)*^

Furthermore, representatives from all participating organisations recognised that, while many initiatives successfully operationalised health equity, structural discrimination continued to contribute to the marginalisation of some groups and to persistent health inequities. The framing of equity in assessing needs might inadvertently result in some groups remaining underserved and further disadvantaged.

### Strategies to better implement health equity

#### Networking

A key strategy for addressing challenges such as poor communication and unstable leadership is the development of strong, transparent networks. Public health experts identified collaboration with local services as a critical enabler in advancing health equity. These community-based networks and intersectoral partnerships were not merely supportive structures, they were perceived to be foundational to equity-oriented work.

Participants emphasised that, although coordinating diverse stakeholders can be complex, sustained collaboration depended on long-term relationship-building and trust. As one public health leader noted,

*Networking among diverse stakeholders is essential*,* but it requires ongoing effort and mutual trust.*^*(PH2)*^

Others reinforced the centrality of networked systems in supporting equity efforts:

*Without a strong network*,* everything falls apart.*^*(PH5)*^

*The system has to work in a network.* ^*(AI3)*^

Building and maintaining these networks was not perceived as a one-time effort; it was an ongoing process that often relied heavily on the interpersonal skills, experience, and commitment of the individuals involved.

#### Long-term system led initiatives

One NGO emphasised that meaningful change stems from organisational culture, noting that long-term initiatives provided the necessary space for equity principles to become embedded within institutional infrastructure and internalised by staff at all levels.

*it’s not just about having the right policies on paper*,* real change happens when equity becomes part of the culture*,* something that shapes how we work*,* think and act everyday.*^*(NGO2)*^

This perspective was echoed by participants from several public health institutions and academic institutions who stressed the importance of sustained, system-level efforts. They argued that health equity should not be viewed as an optional or time-limited objective, but rather as a core organisational value integrated into the very foundation of practice.

*Equity isn’t something you do on the side*,* it has to be part of how the whole system works*,* every day.*^*(AI2)*^

## Discussion

This study aimed to explore the complexity and variability in how health equity is understood, framed, and operationalised across different health-related sectors in the Swiss context. While health equity is widely recognised as a vital public health objective [5, 21, 22], our findings revealed notable gaps between conceptual understanding and practical implementation, across academic, public health, foundations and NGO settings.

A central finding was the variation in conceptual clarity, a theme echoed in previous studies [6, 21–25]. Academic institutions often demonstrated a nuanced understanding of equity, clearly distinguishing it from equality and diversity. However, this depth of understanding did not always translate into concrete institutional action. Several participants acknowledged that health equity is frequently reduced to symbolic gestures or theoretical commitments. In contrast, NGOs and local public health institutions, though sometimes less theoretically precise, they provided more tangible examples of equity being embedded into day-to-day practices. This divergence suggests that shared definitions alone are insufficient; what appears to matter more is how equity is structurally and culturally integrated within organisations [26].

Moreover, this study found how equity is framed, as a moral, political, legal, and/or economic issue, varied not only between organisations but also within them. These varied distinctions shape which issues are prioritised, which populations are considered, and how resources are allocated, a challenge that is also reflected in the wider health literature [27]. The tendency to narrow equity to certain groups illustrates how institutional blind spots can perpetuate exclusion even within equity-driven agendas.

Participants across all sectors pointed to a lack of collaboration, limited data infrastructure, and instability in political leadership as major barriers to advancing health equity. The concept of *“Gärtchendenken”* (akin to silo thinking) captures how cultural and institutional fragmentation can at the same time lead to individual success of an isolated activity and restrict shared learning or coordinated action. Without robust, transparent networks, knowledge exchange is constrained and efforts become duplicated or disconnected [28].

While this phenomenon is described in the Swiss context, challenges of siloed governance are widely recognised in the broader Health in All Policies (HiAP) literature. HiAP emphasises cross-sectoral cooperation, yet its implementation has repeatedly been hindered by fragmented institutional mandates, competing priorities, and weak coordination structure [29, 30]. These barriers are not unique to Bern, Switzerland but reflect a global difficulty in embedding health equity considerations into diverse policy domains. Institutional strategies that integrate equity into decision-making, foster transparent collaboration, and sustain partnerships across sectors are therefore critical for translating HiAP principles into durable action [20, 29, 30].

The findings highlight the absence of reliable, relevant data that could be used to promote health equity evaluation. While some organisations collect pertinent indicators, most lack consistent frameworks or criteria to evaluate equity outcomes, which is often seen in the wider health literature [31, 32]. As a result, even well-intentioned interventions remain largely unevaluated, which may reduce the visibility and accountability of equity efforts. Other international studies have also highlighted that challenges in measuring health equity are often closely linked to limited equity relevant data collection [32].

Policy discontinuity and organisational leadership turnover were also repeatedly cited as destabilising factors. Health equity initiatives often depend on individual champions rather than being institutionally embedded, leading to fragility in progress when personnel or priorities shift [33]. This overreliance renders efforts highly vulnerable, particularly when key personnel depart or organisational focus changes, momentum is frequently lost, and gains are either stalled or reversed [34]. This fragility highlights a deeper structural problem, as there are no lasting, organisation-wide systems in place to ensure that health equity remains a consistent priority [31]. However, as highlighted in the current literature, there is no *“one size fits all”* approach to advancing health equity and decisions about what best suits one organisation may vary depending on which components require the most attention depending on an organisation`s internal and external environment [34].

Despite these challenges, participants identified actionable strategies to advance health equity. Strong, community-based networks and cross-sector partnerships were described as foundational for achieving lasting progress. These networks provide not only operational support but play a critical role in embedding equity principles into local practice, building trust and increasing resilience against leadership changes. However, as noted, such collaboration demands consistent effort and skilled relationship-building. As highlighted within the health equity literature, communities and community-driven actions that promote health are essential components in promoting health equity [26].

Equally important is the implementation of long-term, system-led initiatives. Organisations that treated equity as a core value rather than a project-specific goal reported deeper integration of equity into their culture and infrastructure. With all participants highlighting that real change happens when equity becomes part of the culture. This is reflected in research highlighting that public health efforts seeking to reduce disparities and promote equity must be inclusive to reach their full potential [35]. Embedding equity in this way requires continuous reflexivity, political will, and sustained investment not only in programs but in the people and structures that support them.

### Limitations and future research

This study provides valuable insights into how health equity is conceptualised and operationalised within a diverse range of health-related organisations in Switzerland. However, several limitations should be acknowledged. First, although purposive sampling allowed for inclusion of diverse organisational perspectives (academia, public health, foundations, and NGOs), participants held leadership, coordination, or specialist roles, and their views may reflect institutional or strategic perspectives rather than those of frontline practitioners. Future research could broaden the participant base to include operational staff, who may offer different insights on equity implementation. Second, the study focused exclusively on organisational actors and did not capture the perspectives of individuals or communities who are the intended beneficiaries of equity-driven programmes. Exploring these perspectives, particularly from underserved or marginalised groups, would help identify potential mismatches between institutional goals and lived experiences. Third, as this study provides an in-depth exploration of the Swiss context, its findings may not generalise to countries with different health system structures, political cultures, or historical approaches to equity. Nonetheless, themes such as conceptual ambiguity, data limitations, and structural barriers may be relevant across comparable high-income settings, and cross-national comparative studies could further illuminate how structural and cultural factors shape organisational responses. Finally, while interviews provided rich interpretive insights, subsequent research could strengthen findings through data and technique triangulation. For example, integrating observational methods within organisations would capture day-to-day practices and decision-making, complementing interview data and reducing reliance on self-reported accounts [36].

## Conclusion

Advancing health equity requires not only a shared conceptual understanding but system-wide commitment embedded in institutional culture and structurally aligned. In the Swiss context, characterised by a decentralised health system and strong cantonal autonomy, bridging the gap between theory and action requires tackling structural barriers, improving coordination across sectors, and building resilient, community-networks. Without political stability, solid data, and clear metrics, equity efforts risk fragmentation. Lasting progress requires embedding equity in daily practice and measuring impact through long-term collaboration.

## Supplementary Information

Below is the link to the electronic supplementary material.

Supplementary Material 1

Supplementary Material 2

## Data Availability

Participants did not give consent to share full datasets to protect their anoynmity. De-identified excerpts relevant to the study findings are available from the corresponding author on reasonable request.

## References

[1] European Parliament. Directorate-General for parliamentary. In: Research S, Scholz N, editors. Addressing health inequalities in the European Union – Concepts, action, state of play – In-depth analysis. Publications Office; 2020.

[2] Pan American Health Organization (PAHO). Health Equity n.d [Available from: https://www.paho.org/en/topics/health-equity]

[3] Agency UHS, Health equity and the UK Health Security Agency. 2022 [Available from: https://ukhsa.blog.gov.uk/2022/10/19/health-equity-and-the-uk-health-security-agency/]

[4] Ministry of Health. Achieving equity in health outcomes: highlights of important National and international papers. Wellington: Ministry of Health.; 2018.

[5] Prentice KR, Beitelshees M, Hill A, Jones CH. Defining health equity: A modern US perspective. iScience. 2024;27(12):111326. 39640575 10.1016/j.isci.2024.111326 PMC11617406

[6] Young E. Health equity outlook report. 2024.

[7] National Academies of Sciences E, and Medicine. In: Negussie Y, Geller A, A, editors. Communities in action: pathways to health Equity. Baciu A. Washington, DC: National Academies; 2017. 28418632

[8] Braveman P, Gruskin S. Defining equity in health. J Epidemiol Community Health. 2003;57(4):254–8. 12646539 10.1136/jech.57.4.254 PMC1732430

[9] Browne AJ, Varcoe CM, Wong ST, Smye VL, Lavoie J, Littlejohn D, et al. Closing the health equity gap: evidence-based strategies for primary health care organizations. Int J Equity Health. 2012;11:59. 23061433 10.1186/1475-9276-11-59 PMC3570279

[10] Abiétar DG M-GM, Aguiló E, Sánchez-Valdivia N. Insights on health policies from a political philosophy perspective. J Epidemiol Community Health. 2025;79(4):311–35. 39532394 10.1136/jech-2023-220568

[11] Organization WH. Governance for health equity: taking forward the equity values and goals of health 2020 in the WHO European region. WHO Regional Office for Europe; 2019.

[12] Yusuf J, D’Souza NJ AT, Caldwell H, Meaghan Sim S, Embrett M, Kirk FL. Exploring health equity integration among health service and delivery systems in Nova scotia: perspectives of health system partners. Int J Equity Health. 2024;23(1):171. 39187882 10.1186/s12939-024-02256-7 PMC11345956

[13] Mohajer-Bastami A, Moin S, Sweetman B, Ahmed AR, Head M, Gelber E, et al. A comparison of the united kingdom’s and switzerland’s healthcare financing systems for achieving equity and efficiency goals. Swiss Med Wkly. 2025;155:4101. 40063423 10.57187/s.4101

[14] Frahsa A, Farquet R, Bayram T, De Araujo L, Meyer S, Sakarya S, et al. Experiences with health care services in Switzerland among immigrant women with chronic illnesses. Front Public Health. 2020;8:553438. 33194954 10.3389/fpubh.2020.553438 PMC7608491

[15] Tzogiou C, Boes S, Brunner B. What explains the inequalities in health care utilization between immigrants and non-migrants in switzerland? BMC Public Health. 2021;21(1):530. 33736623 10.1186/s12889-021-10393-9 PMC7977586

[16] Wehrli S, Dwyer AA, Baumgartner MR, Lehmann C, Landolt MA. Lower healthcare access and its association with individual factors and health-related quality of life in adults with rare diseases in Switzerland. Int J Public Health. 2024;69–2024. 10.3389/ijph.2024.1607548 PMC11461209 39386998

[17] Malterud K, Siersma VD, Guassora AD. Sample size in qualitative interview studies: guided by information power. Qual Health Res. 2016;26(13):1753–60. 26613970 10.1177/1049732315617444

[18] Braun VaC V. (2022). London Sage. Thematic analysis: a practical guide. London Sage. 2022.

[19] Braun V, Clarke V, Hayfield N. Terry thematic analysis. In: Liamputtong P, editor. Handbook of research methods in health social sciences Singapore. Springer Singapore. 2019:p 843–60.

[20] Kickbusch I, Buckett K. Implementing health in all policies: Adelaide 2010. Adelaide: Department of Health, Government of South Australia; 2013.

[21] Lewis CL, Yan A, Williams MY, Apen LV, Crawford CL, Morse L, et al. Health equity: a concept analysis. Nurs Outlook. 2023;71(5):102032. 37683597 10.1016/j.outlook.2023.102032

[22] Organization WH. Health Equity. World Health Organization, [Available from: https://www.who.int/health-topics/health-equity#tab=tab_1]

[23] Amri M, Enright T, O’Campo P, Di Ruggiero E, Siddiqi A, Bump JB. Investigating inconsistencies regarding health equity in select world health organization texts: a critical discourse analysis of health promotion, social determinants of health, and urban health texts, 2008–2016. BMC Global Public Health. 2024;2(1):81. 39681940 10.1186/s44263-024-00106-w PMC11622997

[24] Braveman PA, Kumanyika S, Fielding J, Laveist T, Borrell LN, Manderscheid R, et al. Health disparities and health equity: the issue is justice. Am J Public Health. 2011;101(Suppl 1):S149–55. 21551385 10.2105/AJPH.2010.300062 PMC3222512

[25] Organization WH. Health Systems in Action: Switzerland. Geneva. 2024.

[26] Baciu A, Negussie Y, Geller A, Weinstein JN. The root causes of health Inequity. Communities in action: pathways to health equity. Washington, DC: National Academies Press (US); 2017. Chapter 3. 28418632

[27] Organization WH. Closing the gap in a generation: health equity through action on the social determinants of health. Final report of the commission on social determinants of health. Geneva: World Health Organisation; 2008. 10.1016/S0140-6736(08)61690-6 18994664

[28] Chen AM. Barriers to health equity in the united States of america: can they be overcome? Int J Equity Health. 2025;24(1):39. 39920763 10.1186/s12939-025-02401-w PMC11806735

[29] Holt DHFK, Tjørnhøj-Thomsen T, Clavier C. Opening the ‘black box’ of health in all policies: a realist analysis of the implementation of a municipal health in all policies strategy. Scand J Public Health. 2018;46(6):613–23.29226798

[30] Shankardass K, Muntaner C, Kokkinen L, Shahidi FV, Freiler A, Oneka G, et al. The implementation of health in all policies initiatives: a systems framework for government action. Health Res Policy Syst. 2018;16(1):26. 29544496 10.1186/s12961-018-0295-z PMC5856219

[31] Caldwell HAT, Yusuf J, Carrea C, Conrad P, Embrett M, Fierlbeck K, et al. Strategies and indicators to integrate health equity in health service and delivery systems in high-income countries: a scoping review. JBI Evid Synth. 2024;22(6):949–1070. 38632975 10.11124/JBIES-23-00051 PMC11163892

[32] Barcellona C, Mariñas YB, Tan SY, Lee G, Ko KC, Chham S, et al. Measuring health equity in the ASEAN region: conceptual framework and assessment of data availability. Int J Equity Health. 2023;22(1):251. 38053205 10.1186/s12939-023-02059-2 PMC10696689

[33] van Roode T, Pauly BM, Marcellus L, Strosher HW, Shahram S, Dang P, et al. Values are not enough: qualitative study identifying critical elements for prioritization of health equity in health systems. Int J Equity Health. 2020;19(1):162. 32933539 10.1186/s12939-020-01276-3 PMC7493313

[34] Doherty JA, Johnson M, McPheron H. Advancing health equity through organizational change: perspectives from health care leaders. Health Care Manage Rev. 2022;47(3):263–70. 34456273 10.1097/HMR.0000000000000326 PMC9162074

[35] Hood SCB, Baker K. Culturally informed community engagement: implications for inclusive science and health equity. Research Triangle Park: RTI; 2023. 37289927

[36] Carter N, Bryant-Lukosius D, DiCenso A, Blythe J, Neville AJ. The use of triangulation in qualitative research. Oncol Nurs Forum. 2014;41(5):545–7. 25158659 10.1188/14.ONF.545-547

