# Supplementary material for: From rhetoric to reality: organisational practices of health equity in Switzerland
Source: Int J Equity Health. 2025 Nov 28;24:336. doi: 10.1186/s12939-025-02688-9 (PMC12661817; doi:10.1186/s12939-025-02688-9)
Supplement: Supplementary file 1 — Supplementary Material 1 [file 12939_2025_2688_MOESM1_ESM.docx]

***Exploring the role of health equity in the Swiss health sector***

**INTERVIEW QUESTIONS**

1. Please tell us a little about your role and the organisation you work for, as well as your interest in this study

| Enquiry questions | Maintence questions | Themes |
| --- | --- | --- |
| - How long have you been working there? - What exactly is the name of your department/ area of work? - How long have you been responsible for this topic? | Can you describe that in more detail? | Role in the organisation |
| - Why did you respond to my contact request? | Can you explain that in more detail? | Interest and motivation |

1. What do you specifically associate with the term’s health equity and health equality?

| Enquiry questions | Maintence questions | Themes |
| --- | --- | --- |
| - How would you define health equity? - How would you define health inequality? - In your opinion, what are the differences/similarities between these concepts? | Can you explain that in more detail? | Understanding and definitions |
| - Are there any instances where they have been used interchangeably? - Are there challenges with distinguishing between these concepts within your organisation? - If yes, in what way | In what way?  Can you give me an example/s? | Conceptual/ Operational differences |

1. What significance does health equity have for your organisation?

| Enquiry questions | Maintenance issues | Themes |
| --- | --- | --- |
| - What makes health equity important for you? | Please explain in more detail  In what way? | Personal meaning |
| - What makes it important to your/your organisation? think it’s important? | In what way? | Meaning for the organisation |
| - What makes health equity important for the wider community? | In what way | Meaning for the community |
| - What resources are reserved or required to advance/improve health equity within your organization? | To what extent  Can you explain that in more detail | Resources |
| - How much is health equity a priority within your organisation? - How long as health equity been a prominent theme within your organisation? - How would you describe your organisational readiness to integrate and/or advance health equity within your health service? | To what extent  Please go into more detail | Commitments/obligations |
| - Is health equity an explicit consideration in the goals and objectives for your health service and/programs run by your organisation? - What are you currently focussing on? - Can you share examples of programs or projects that have successfully addressed health disparities? | In what way?  To what extent?  Give examples | Opportunities |

1. What challenges or barriers do you see particularly in your context in relation to the topic?

| Enquiry questions | Maintenance issues | Themes |
| --- | --- | --- |
| - What/if any issues does integrating health equity have (general)? - What types of challenges does integrating health equity into your organisation/programs pose? - Do different programs/ services pose different challenges? | In what way?  Can you give examples? | Organisational barriers |
| - What are the barriers to integrate/ advance health equity in the wider communities in Bern | In what way? | Community barriers |
| - What strategies/solutions do you implement to overcome these challenges? | Please give examples. | Strategies/solutions |
| - What would you recommend to improve integrating health equity in your own organisation? - What would you recommend to improve integrating health equity to other similar organisations? | Can you explain in more detail? | Recommendations |

1. How do you make health equity measurable? How do you measure success in this context?

| Enquiry questions | Maintenance issues | Themes |
| --- | --- | --- |
| - Is health equity assessed or evaluated within - your organisation?   -service  -programs   - If yes, how is it measured? - Are they measured using specific measures/ health equity measures | In what way?  Can you give examples? | Assessment/evaluation |
| - If yes, how are these findings used? - If no, What recommendations would you make to integrate assessment or evaluation measures? | Please explain in more detail | Results/findings |
| - Are these findings shared internally within the organisation - Are these findings shared with similar organisations? - Are any official statistics or findings reported? | In what way? | Dissemination of information |
| - What/if any are the obstacles/challenges that prevent collecting health equity data? | Please explain in more detail | Barriers/challenges |
| - Could the assessment process be improved? - How could this be implemented? | In what way? | Recommendations |
| - Do you think health equity can be measured /should be measured? | To what extent? | Personal opinion |

6. Who are other important players for health equity?

| Enquiry questions | Maintenance issues | Themes |
| --- | --- | --- |
| - Who else do you think would contribute to this topic | Can you explain in more detail? | Health services/stakeholders |
| - Do health services work together on this topic?   -transparency  -findings  -experience | In what way? | Cooperation |
| - If no, what do you think could be done to encourage services to work together on the topic of health equity? | Can you explain in more detail? | Challenges |

1. What recommendations do you have for realising equal health opportunities locally?

| Enquiry questions | Maintenance issues | Themes |
| --- | --- | --- |
| - What policy changes would you recommend to improve health equity in Switzerland? - How can federal and cantonal governments better support efforts to reduce health disparities? | In what way?  How would this be implemented? | Recommendations |

**Closing question**

- Is there anything else you would like to share about health equity that we haven’t discussed?

Thank you for your time and participation
