# Supplementary material for: From rhetoric to reality: organisational practices of health equity in Switzerland
Source: Int J Equity Health. 2025 Nov 28;24:336. doi: 10.1186/s12939-025-02688-9 (PMC12661817; doi:10.1186/s12939-025-02688-9)
Supplement: Supplementary file 2 — Supplementary Material 2 [file 12939_2025_2688_MOESM2_ESM.docx]

**Appendix 2: Reflexivity statement**

Given the multidisciplinary background and expertise of the author team, reflexivity was actively incorporated throughout the research process to ensure transparency and awareness of potential influences on the study. The first author brings experience in psychology, sociology, and public health, alongside professional exposure within the Swiss health system. The co-author brings in expertise in psychology and the last author and supervisor contributes expertise in community health, social determinants of health, political science and qualitative research methods, with long-standing engagement in Swiss and international public health contexts.

Reflexive practices were applied at multiple stages:

1. **Conceptual framework selection** Our political and professional experiences shaped the design of this study. We had been in contact with different actors about a proposed project on healthy and equitable urban spaces during which several people expressed interest to initiating exchange about health equity or even suggested to meet and be interviewed. At the same time, the first author, trained in the UK, and the last author, trained in Germany, recognized how terms were used differently in their contexts as well as depending on conversations taking place in English or German with our partners in the Swiss context. This made us decide to explore understandings and organisational practices in the Swiss context in more detail. Our background also shaped the working definition of equity and equality applied, the choice of theoretical frameworks and research questions, prompting reflection on assumptions and potential biases. Engagement with partner organisations revealed both the opportunities and challenges of implementing health equity, while their strong engagement with the theme underscored its importance, reinforcing the need to align theory with practice.
2. **Data generation:** Awareness of our specific positionality guided the approach to interviews and observations, asking participants for their working definitions and understandings, taking time and room for examples and clarifications as well as discussions during interviews, fostering sensitivity to participants’ perspectives and maintaining a non-judgmental stance.

Through this reflexive approach, we aimed to enhance the rigor and credibility of the study while acknowledging and embracing the inevitable influence of researchers’ backgrounds on the research process.
